# New Books

**Published:** 2008-08

**Acid Rain in the Adirondacks: An Environmental History**

Jerry Jenkins, Karen Roy, Charles Driscoll, Christopher Buerkett

Ithaca, NY:Cornell University Press, 2007. 256 pp. ISBN: 978-0-8014-4651-1, $65

**A Dictionary of Biology, 6th ed.**

Elizabeth Martin, Robert S. Hine, eds.

New York:Oxford University Press, 2008. 736 pp. ISBN: 978-0-19-920462-5, $17.95

**America’s Food: What You Don’t Know About What You Eat**

Harvey Blatt

Cambridge, MA:MIT Press, 2008. 3874 pp. ISBN: 978-0-262-02652-9, $29.95

**Apoptosis and Cancer**

Gil Mor, Ayesha Alvero, eds.

New York:Springer, 2008. 350 pp. ISBN: 978-1-58829-457-9, $99.50

**Biogeochemical Cycles in Globalization and Sustainable Development**

Vladimir F. Krapivin, Costas Varotsos

New York:Springer, 2008. 562 pp. ISBN: 978-3-540-75439-8, $269

**Choosing Safety: A Guide to Using Probabilistic Risk Assessment and Decision Analysis in Complex, High Consequence Systems**

Michael V. Frank

Washington, DC:RFF Press, 2008. 230 pp. ISBN: 978-1-933115-53-5, $85

**Contaminant Geochemistry: Interactions and Transport in the Subsurface Environment**

Brian Berkowitz, Ishai Dror, Bruno Yaron

New York:Springer, 2008, 412 pp. ISBN: 978-3-540-74381-1, $189

**Diagnosis: Mercury: Money, Politics, and Poison**

Jane M. Hightower

Washington, DC:Island Press, 2008. 288 pp. ISBN: 978-1-59726-395-5, $24.95

**Environmental History of the Rhine–Meuse Delta**

P.H. Nienhuis

New York:Springer, 2008. 642 pp. ISBN: 978-1-4020-8211-5, $239

**Environmental Policy Convergence in Europe: The Impact of International Institutions and Trade**

Katharina Holzinger, ed.

New York:Cambridge University Press, 2008. 288 pp. ISBN: 978-0-521-88881-3, $90

**Essential Concepts in Toxicogenomics**

Donna L. Mendrick, William B. Mattes, eds.

Totowa, NJ:Humana Press, 2008. 324 pp. ISBN: 978-1-5882-9638-2, $99.50

**Global Catastrophic Risks**

Nick Bostrom, Milan M. Cirkovic, Martin J. Rees, eds.

New York:Oxford University Press, 2008. 576 pp. ISBN: 978-0-19-857050-9, $50

**Green Urbanism Down Under: Learning from Sustainable Communities in Australia**

Timothy Beatley with Peter Newman

Washington, DC:Island Press, 2008. 368 pp. ISBN: 978-1-59726-411-2, $70

**Innovations and the Environment**

Yoram Krozer

New York:Springer, 2008. 202 pp. ISBN: 978-1-84800-196-1, $119

**Investigating Science Communication in the Information Age: Implications for Public Engagement and Popular Media**

Richard Holliman, Elizabeth Whitelegg, Eileen Scanlon, Sam Smidt, Jeff Thomas, eds.

New York:Oxford University Press, 2008. 320 pp. ISBN: 978-0-19-955266-5, $39.95

**Lake Effect: Two Sisters and a Town’s Toxic Legacy**

Nancy A. Nichols

Washington, DC:Island Press, 2008. 208 pp. ISBN: 978-1-59726-084-8, $24.95

**Particularly Sensitive Sea Areas: The IMO’s Role in Protecting Vulnerable Marine Areas**

Markus J. Kache

New York:Springer, 2008. 376 pp. ISBN: 978-3-5407-8778-5, $159

**Protection from Exposure to Second-hand Smoke: Policy Recommendations**

World Health Organization

Geneva:WHO Press, 2007. 50 pp. ISNB: 978-924-556341-9, $15

**Seasonal Forecasts, Climatic Change and Human Health**

Madeleine C. Thomson, Ricardo Garcia-Herrera, Martin Beniston, eds.

New York: Springer, 2008. 234 pp. ISBN: 978-1-4020-6876-8, $189

**The Chemical Components of Tobacco and Tobacco Smoke**

Alan Rodgman, Thomas A. Perfetti

Boca Raton, FL:CRC Press, 2008. 928 pp. ISBN: 978-1-420-07883-1, $299.95

**The Continental-Scale Greenhouse Gas Balance of Europe**

Johannes Dolman, Riccardo Valentini , Annette Freibauer, eds.

New York:Springer, 2008. 390 pp. ISBN: 978-0-387-76568-6, $129

**The Design of Climate Policy**

Roger Guesnerie, Henry Tulkens, eds.

Cambridge, MA:MIT Press, 2009. 408 pp. ISBN: 978-0-262-07302-8, $38

**The Earth’s Atmosphere: Its Physics and Dynamics**

Kshudiram Saha

New York:Springer, 2008. 370 pp. ISBN: 978-3-540-78426-5, $219

**The Music of Life: Biology Beyond Genes**

Denis Noble

New York:Oxford University Press, 2008. 176 pp. ISBN: 978-0-19-922836-2, $15.95

